# A new Li/Mg paleothermometer from pteropod shells

**DOI:** 10.1038/s41598-026-55990-z

**Published:** 2026-06-11

**Authors:** N. Keul, D. Garbe-Schönberg, V. Kitidis, G. Langer, K. T. C. A. Peijnenburg

**Affiliations:** 1 Institute of Geosciences, Christian-Albrechts-Universität zu Kiel, Ludewig-Meyn-Str.10, 24118 Kiel, Germany; 2 Plymouth Marine Laboratory, Plymouth, UK; 3 Alfred Wegener Institute, Am Handelshafen 12, 27570 Bremerhaven, Germany; 4 Naturalis Biodiversity Center, P.O. Box 9517, 2300 RA Leiden, The Netherlands; 5 Institute for Biodiversity and Ecosystem Dynamics (IBED), University of Amsterdam, P.O. Box 94248, 1090 GE Amsterdam, The Netherlands

**Keywords:** *Heliconoides inflatus*, Paleoceanography, Proxy development, Trace elemental composition, Paleothermometry, Atlantic Ocean, Climate sciences, Ocean sciences, Solid Earth sciences

## Abstract

Pteropods are promising proxy archives for paleoceanographic reconstructions but remain underexplored. Previous oxygen isotope analyses indicated that *Heliconoides inflatus* calcifies at shallow depths (50–75 m), suggesting its potential to record surface-ocean conditions. Here, we evaluate for the first time the applicability of Li/Mg thermometry in pteropod shells, a temperature proxy widely used in coral studies. We show that, in addition to recording environmental variability through shell carbon and oxygen isotopic composition, *H. inflatus* shells record upper-water temperature conditions, with Li/Mg ratios showing a robust relationship with ambient temperatures at sampling locations. Li/Mg ratios decrease exponentially with increasing temperature, enabling temperature reconstructions with an average precision of ± 1–2 °C. Pteropod shells (species *H. inflatus*, formerly known as *Limacina inflata*) were collected along a latitudinal transect in the Atlantic Ocean (31° N to 38° S), spanning a surface-water temperature gradient of approximately 15 °C. The global distribution and abundance of this annual species in sediments makes it a suitable alternative for paleo-surface temperature reconstruction. The utility of pteropod shells extends further to seasonal paleotemperature reconstruction, as seasonal temperature variability is captured in the elemental ratios from the embryonic to later (adult) sections of the shell. These results, in combination with a basin-scale distribution, make the Li/Mg thermometer in pteropods an exciting new tool in paleoceanography and further cement this group as new proxy archives.

**Supplementary Information:**

The online version contains supplementary material available at 10.1038/s41598-026-55990-z.

## Introduction

Assessing the future impact of anthropogenic CO_2_ emissions and consequent temperature increase on marine ecosystems is difficult, as the complexity of ecosystems cannot be easily replicated in laboratory experiments. Geological records provide long-term evidence of global warming and ocean acidification, along with the responses of marine calcifiers. These records of marine calcifiers reveal past ocean temperatures and chemistry through fossilized calcium carbonate shells, as well as ecological indicators like species richness and net calcification. Multiple proxies have been developed to assess temperatures in the upper ocean, such as Mg/Ca and δ^18^O in planktonic foraminifera^1,2^, Sr/Ca in corals^3^, as well as clumped isotope thermometry in coccoliths^4^, foraminifera^5^, and molluscs^6,7^. Additional approaches include organic geochemical proxies such as alkenones in sediments^8^ and TEX₈₆^9^.

Despite the growing use of trace-element proxies in marine calcifiers, geochemical investigations of trace element incorporation in pteropods remain scarce. Apart from an early study examining elemental incorporation in pteropod shells^10^, no comprehensive analyses of multiple trace metals in this group have been conducted. In contrast, several studies on other calcifying taxa have explored relationships between environmental conditions and shell geochemistry. For example, there were several investigations of the trace elemental composition of aragonitic gastropods such as *Turbo torquatus*^11^ and *Patella caerulea*^12^*,* or on modern brachiopods^13^*.* Their results highlight both the promise and uncertainty of trace-element proxies in aragonitic organisms and underscore the need to evaluate their applicability.

Another prominent and relatively recent proxy for reconstructing ambient seawater temperatures is the Li/Mg ratio in aragonitic corals and foraminifera^14–16^. This thermometer has evolved from an initial exponential calibration, in which skeletal Li/Mg shows a direct relationship with seawater temperature^16,17^ to more refined multi-proxy approaches that combine Li/Mg with Sr/Ca^18–20^. Compared with other geochemical proxies, Li/Mg is considered to be less influenced by organismal physiology or skeletal heterogeneity⁷, making it a promising candidate for application to pteropod shells using laser ablation inductively coupled plasma mass spectrometry (LA-ICP-MS).

Pteropods are holoplanktonic marine gastropods found in both epipelagic and mesopelagic zones, from the sea surface to depths exceeding 1000 meters^21^. While they have been studied less extensively than other calcifying plankton (e.g. foraminifera, coccolithophores and calcareous dinoflagellates) as potential oceanographic proxy archives, various features of their biology make them promising candidates. The shells of thecosome pteropods are composed of aragonitic calcium carbonate, which, in combination with their relatively thin shells, makes them particularly sensitive recorders of seawater carbonate chemistry and environmental conditions^22^. Their approximately annual life cycle^23,24^ may yield a more integrative proxy record across seasons compared to planktonic foraminifera, which are characterized by a shorter life span (ca. 1 month)^25^. Even though pteropods are known to carry out diel vertical migrations and some species are found at great depths^21^, the actual calcification depth of most species is probably much shallower (50–250 m depth^24,26,27^). In a previous study using the same samples that are investigated here, we were able to establish a shallow calcification depth of ca. 50–75m for *H. inflatus* in the Atlantic^27^. In the same study we showed that pteropod shells are excellent recorders of climate change, as carbonate ion concentration and temperature in the upper water column have dominant influences on pteropod shell carbon and oxygen isotopic composition^27^.

While the previous work emphasized isotopic responses to environmental parameters, the present study extends these observations by examining trace-element ratios in the same specimens, in order to assess the potential of the Li/Mg thermometer in pteropod shells and to present trace elemental ratios of *Heliconoides inflatus* (namely Li/Ca, Mg/Ca, and calibrations for the Li/Mg thermometer). Our material was collected along a meridional transect in the Atlantic Ocean, ranging from 31°N to 38°S, spanning a surface-water temperature gradient of approximately 15 °C. The studied species, *H. inflatus*, occurs in high abundance in sediments worldwide, for instance, in the Atlantic (Caribbean Sea^28^, Cariaco Basin^29^), Pacific (North West Shelf of Australia^30^) and the Indian Ocean (off the Maldives^31^). Because pteropod shells are formed over several months, the geochemical composition of a fully grown shell likely integrates environmental conditions experienced throughout much of the organism’s lifespan rather than representing instantaneous conditions at the time of collection. Consequently, contemporaneous in-situ temperatures are used here as an approximation of the characteristic upper-water temperature regime at each station rather than as exact calcification temperatures. We expect that establishing a new temperature proxy for pteropods allows the disentanglement of temperature from secondary influences on the stable isotopic composition.

## Results

### Surface distribution of oceanographic parameters in the study area

**Fig. 1:**
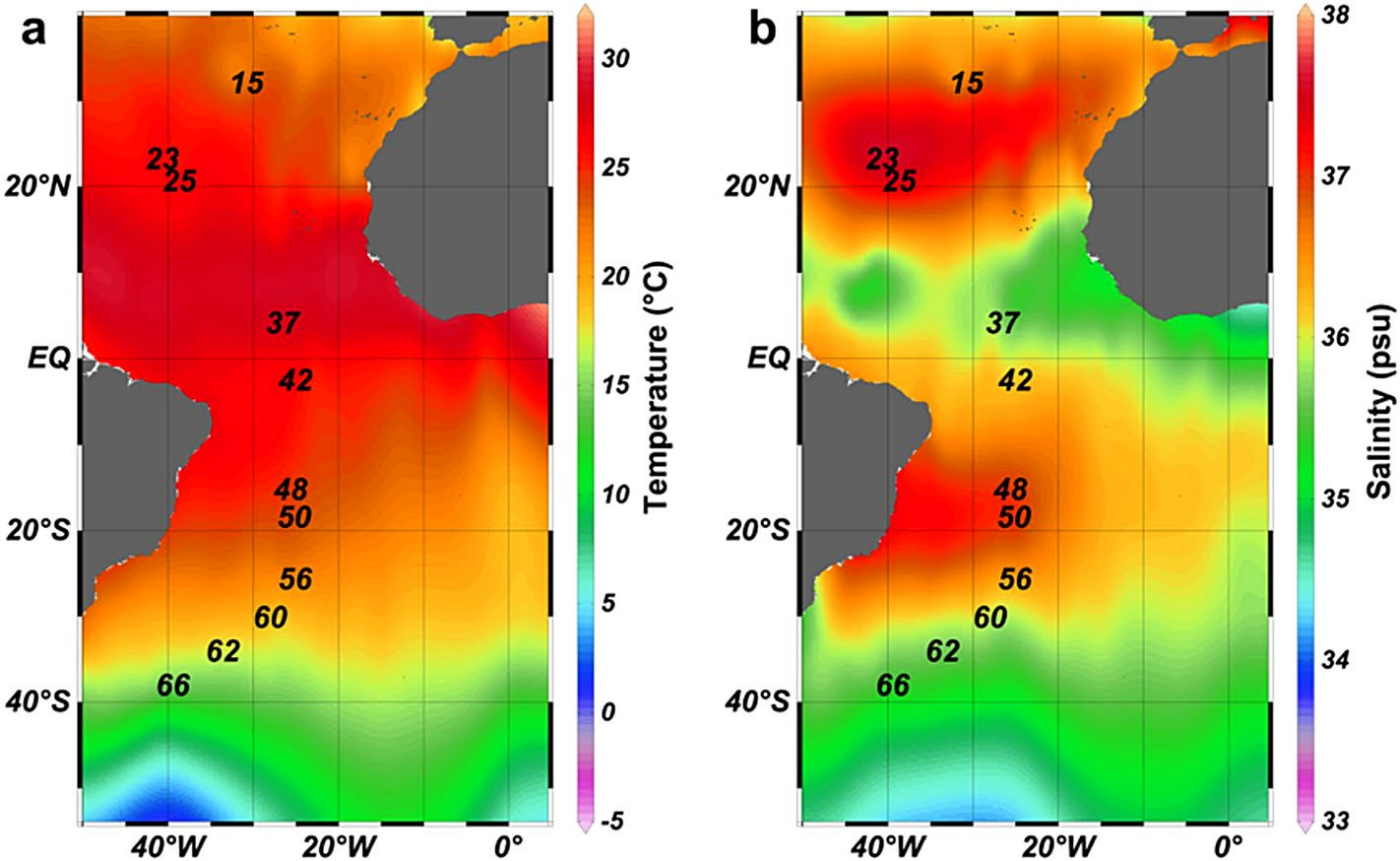
Distribution plots of (**a**) temperature (° Celsius) and (**b**) salinity (psu) in surface waters in the Atlantic Ocean. Only cruise stations from which pteropods were sampled for this study are shown. Data are derived from the World Ocean Database^32^ and represent average values during the months of the cruise (October and November) between 1986 and 2011. The software Ocean Data View (v. 4.6.3, http://odv.awi.de) was used to generate these maps^33^. Modified after Fig. 1 in Keul et al.^27^

Zooplankton, water samples and in-situ data were collected during the Atlantic Meridional Transect Cruise 22 (AMT22), which took place from October 19 to November 16 in 2012, between 31°N and 38°S. The warmest surface temperatures, up to 30 °C, occurred in October/ November just north of the equator (around 10°N; Figs. S1d, 1a). Temperature decreased gradually both north and south of this maximum and reached approximately 12°C at 38°S, where our southernmost station was located (Fig. S1d). The surface salinity distribution mimicked this general latitudinal zonation (Fig. 1b), however, at the latitude of the temperature maximum, low salinities of (< 36) prevailed. Highest surface salinities (> 37) occurred around 25° N and 20° S west of 30° W.

### Trace elemental composition of pteropod shells (Li/Ca and Mg/Ca)

Individual pteropods were manually sorted from bulk zooplankton net samples. The trace elemental (TE) composition of pteropod shells was determined by LA-ICP-MS (laser ablation inductively coupled mass spectrometry). Individual pteropod TE/Ca ratios vary over the latitudes (and temperatures) analyzed (see Table 1, Table S1 and Fig. S1a–c). Average pteropod Li/Ca values range from 3.55 to 6.36 µmol/mol (Table 1, Fig. S1a), with spot measurements ranging from 1.66 to 11.69 µmol/mol (Table S1). Variability in pteropod Li/Ca, expressed as the relative standard deviation (RSD; ratio of standard deviation to mean, in percent), is on average 23%. The highest values are found at the southernmost station (38.11°S) and the lowest values at the northernmost station (32.02°N).

**Table 1:** Station location information (Lat.= Latitude, Long= Longitude), Temperature at 50m depth (Temp, in °C), Salinity at 50m depth (Sal.) and average trace elemental composition (Li/Ca in μmol/mol, Mg/Ca in mmol/mol, Li/Mg in μmol/ mmol) with their standard errors (SE).

Station	Lat.	Long.	Temp.	Sal.	Li/Ca	SE	Mg/Ca	SE	Li/Mg	SE
15	32.02	− 30.74	23.22	36.79	6.36	1.78	0.84	0.55	17.21	4.70
23	23.16	− 40.60	26.96	37.43	3.77	0.49	0.33	0.02	13.18	1.84
25	20.67	− 38.56	26.70	37.36	3.55	0.10	0.25	0.01	15.67	0.28
37	4.03	− 26.47	28.26	35.70	3.57	0.14	0.44	0.07	9.37	1.85
42	− 4.62	− 25.00	25.28	36.12	5.47	0.40	0.77	0.38	15.80	1.56
48	− 15.29	− 25.13	23.78	37.23	4.03	0.47	0.30	0.05	15.95	3.89
50	− 18.52	− 25.10	22.79	36.95	5.02	0.12	0.24	0.02	22.38	1.32
56	− 25.75	− 24.99	21.57	36.67	4.03	0.38	0.26	0.03	16.08	3.08
60	− 30.20	− 27.92	19.73	36.16	5.42	0.26	0.33	0.02	17.06	2.21
62	− 34.15	− 33.49	16.73	35.60	6.37	0.60	0.20	0.00	35.29	3.96
66	− 38.11	− 39.33	14.36	35.04	5.43	0.12	0.21	0.02	27.28	0.67

Pteropod Mg/Ca values (Table 1, Fig. S1b) vary on average from 0.20 to 0.84 mmol/mol, while spot measurements range from 0.13 to 3.93 mmol/mol (Table S1), with an average RSD of 37%. The comparatively high intra-shell variability observed for both Li/Ca and Mg/Ca warrants further investigation of intra-shell compositional trends, which is addressed below.

### The Li/Mg thermometer in pteropod shells

**Fig. 2:**
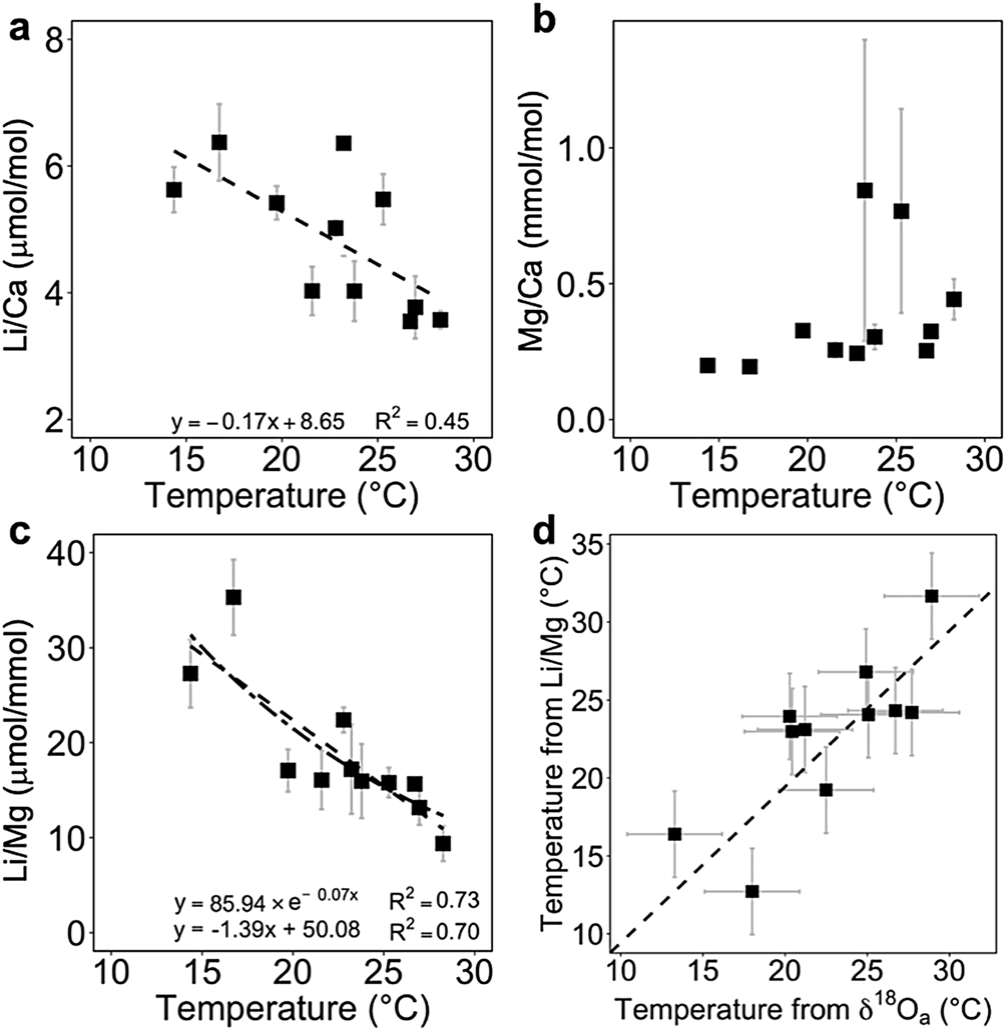
Relationships between Li/Ca, Mg/Ca, and Li/Mg ratios in *H. inflatus* shells and upper-ocean temperature. (**a**) Li/Ca (μmol/mol), (**b**) Mg/Ca (mmol/mol) and (**c**) Li/Mg (μmol/mmol) versus temperature at 50 m depth and (**d**) temperature derived from Li/Mg versus temperature derived from δ^18^O^ 27^. Data points represent station-averaged shell compositions, where each average integrates multiple laser spots per shell and three shells per station. Temperatures correspond to contemporaneous CTD measurements at 50 m depth, which approximate the mean environmental conditions experienced during shell formation rather than instantaneous calcification temperatures. Dashed lines indicate statistically significant linear regressions (p < 0.05), while the dot-dashed line in (c) represents the exponential fit commonly used in Li/Mg paleothermometry. Dashed line in (d) represents the 1:1 line where calculated Li/Mg temperatures would exactly equal δ1⁸O-derived temperatures. Error bars indicate standard errors of station averages; in some cases, these are smaller than the symbols. Data sources: Tables 1, 2. All data points shown represent station averages; no outliers were excluded.

Average pteropod Li/Mg values (Fig. S1c) range from 9.37 to 35.29 µmol/mmol, while spot measurements display a broader range of 2.05–52.64 µmol/mmol, with an average RSD of 38% (Table 1, Table S1, Fig. S1c). While Li/Ca ratios show a weak negative correlation with temperatures at all tested depths between 2 and 75 m (p < 0.05, R^2^ = 0.39—0.45; Table S2a, Fig. 2a), no significant relationship is observed between Mg/Ca and any tested depth (2-300 m, p > 0.05; Table S2b, Fig. 2b).

**Table 2:** (**a**) Linear and (**b**) correlation between average pteropod shell Li/Mg (μmol/mmol) and selected water parameters: temperature (° Celsius) and salinity. Regressions were performed against parameters at specific depths; the R^2^ is reported when *p* < 0.05. *n.s*. indicates non-significant regressions (*p* > 0.05). Regressions for correlations with temperature at 50 m depth are listed above the tables, values in parentheses are the respective standard errors.

Depth (m)	Temperature	Salinity
***a*** **R**^2^ **values for Li/Mg versus (linear correlation)**		
2	0.75	n.s
25	0.69	n.s
50	0.70	n.s
75	0.62	n.s
100	n.s	n.s
150	n.s	n.s
200	n.s	n.s
250	n.s	n.s
300	n.s	n.s
***b*** **R**^2^ **values for Li/Mg versus (exponential correlation)**		
2	0.75	n.s
25	0.71	n.s
50	0.73	n.s
75	0.58	n.s
100	n.s	n.s
150	n.s	n.s
200	n.s	n.s
250	n.s	n.s
300	n.s	n.s

Li/Mg ratios display a significant negative relationship with all tested surface (2- 75 m) temperatures, described by both a linear (p < 0.05, R^2^ = 0.62 (at 75 m)−0.75 (at 2 m)) and an exponential fit (p < 0.05, R^2^ = 0.58 (at 75 m) −0.75 (at 2 m); Table 2, Fig. 2c). Our dataset does not allow discrimination between the two fits; therefore, both relationships are reported, although exponential relationships are more commonly reported in the literature for Li/Mg paleothermometry^16^. The following equations describe the linear and exponential correlations with temperature (T) at 50 m water depth:

1 $${Li}/{{Mg}}\,\left( {\mu {\mathrm{mol/mmol}}} \right) = - 1.39\,\left( { \pm 0.30} \right) \times {\mathrm{T}} + 50.08\,\left( { \pm 7.00} \right)$$

with p < 0.05, R^2^ = 0.70, and

2 $${Li}/{{Mg}}\,\left( {\mu {\mathrm{mol/mmol}}} \right) = 85.94 \, \left( { \pm 27.8} \right) exp^{{\left( { - 0.070 \left( { \pm 0.014} \right) \times {\mathrm{T}} } \right)}}$$

with p < 0.05, R^2^ = 0.73 (Table S2).

We also tested for correlations between trace-element ratios (Li/Ca, Mg/Ca, Li/Mg) and surface salinity across the transect. No significant relationships were observed (p > 0.1: Tables 2, S2), suggesting that salinity variations within our sampling range did not strongly influence Li/Mg incorporation in *H. inflatus* shells. In addition, stable oxygen isotope ratios (δ^1^⁸O) measured on the same set of pteropod samples have previously been shown to provide a reliable proxy for ambient seawater temperature at the depth of shell calcification (ca. 50–75 m)^27^. Consequently, temperatures at 50 m depth were used for the Li/Mg–temperature calibration presented here. Furthermore, temperatures derived from Li/Mg ratios are positively correlated with δ^1^⁸O-derived temperatures (p < 0.05, R^2^ = 0.57; Fig. 2d, Fig. S2), providing independent support for the temperature sensitivity of Li/Mg ratios in pteropod shells.

### Compositional variability of Mg/Ca, Li/Ca and Li/Mg ratios in pteropods

**Fig. 3:**
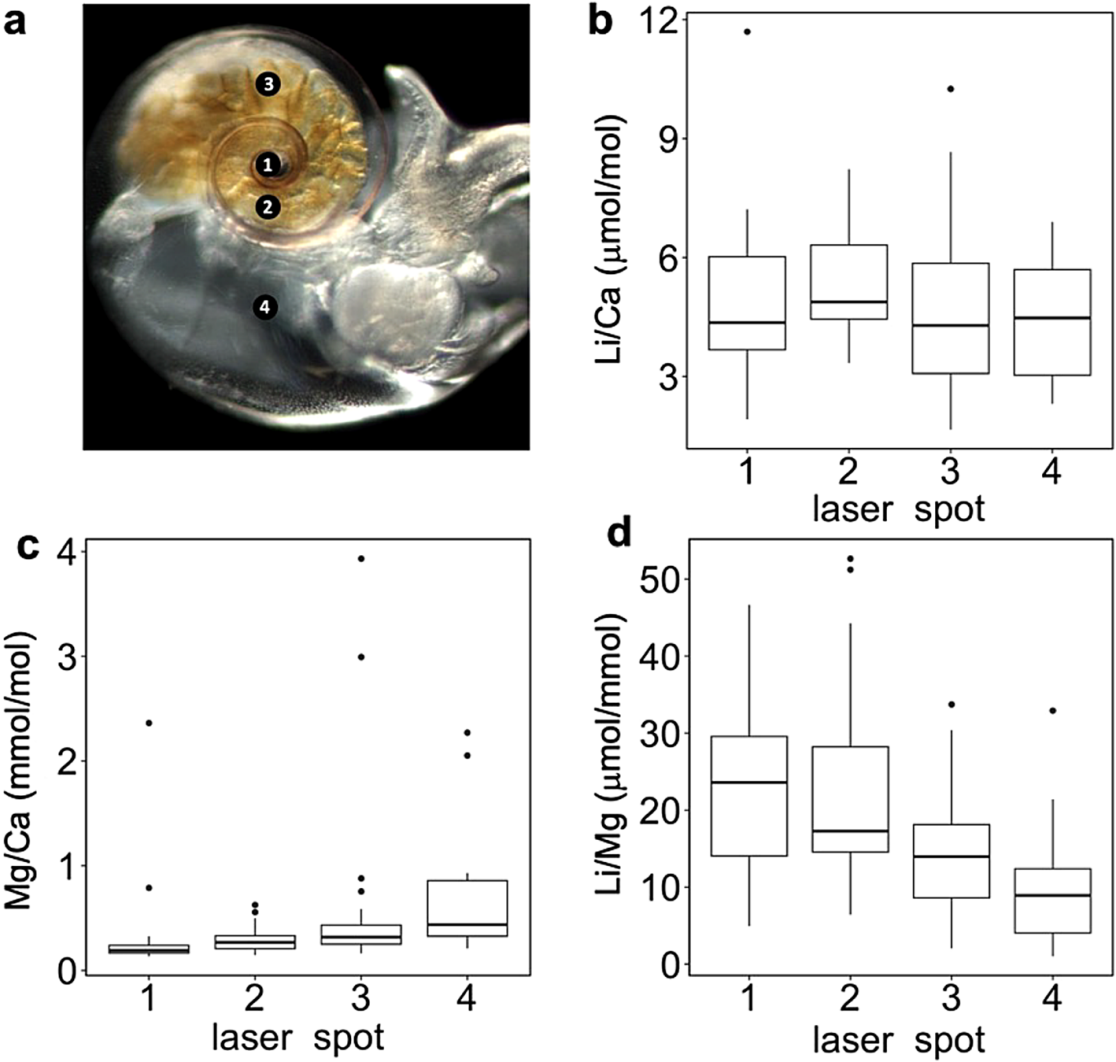
Intra-shell variability of Li/Ca, Mg/Ca, and Li/Mg ratios in *H. inflatus*.** (a)** Photograph of pteropod shell indicating laser-ablation spots. Spots 1 to 4 correspond to embryonic (spot 1) to juvenile to adult shell material (spot 4). (**b**) Li/Ca (μmol/mol), (**c**) Mg/Ca (mmol/mol) and (**d**) Li/Mg (μmol/mmol) measured at laser spots 1–4. Boxplots show interquartile range (IQR) and median values; whiskers extend to 1.5 × IQR. Data points outside this range (if any) are plotted as potential outliers. Data interpretation: Measurements represent sub-shell laser spots, not station averages. Intra-shell variability provides insight into ontogenetic and environmental changes during growth, but quantitative temperature reconstructions should be based on integrated, station-averaged shell compositions rather than on laser spots. Statistical significance between laser spots was assessed using Wilcoxon or Welch’s t-test (p < 0.05). Data source: Table S1. No outliers were excluded.

Trace elemental/Ca ratios from LA-ICP-MS tend to have a much higher variability compared to traditional ICP-MS analysis of whole, acid-dissolved shells, where the intra-test inhomogeneity cannot be resolved. The average RSD of 23% for Li/Ca and 37% for Mg/Ca highlights pronounced compositional variability within individual pteropod shells and motivates a more detailed assessment of intra-shell trends. Laser ablation targets were placed at multiple positions across the shell, ranging from the innermost embryonic shell (spot 1; Fig. 3a) to progressively younger shell material formed later in ontogeny (spots 2–4; Fig. 3a). For clarity, laser spot 1 corresponds to the innermost, embryonic or very early juvenile shell material, while laser spots 2–4 represent progressively younger shell material formed later during ontogeny; this spatial framework is used throughout the Discussion when referring to shell growth stages. No statistically significant difference in median values was detected for Li/Ca ratios between measurements obtained from spot 1 and spot 4 (p > 0.05; Fig. 3b).

In contrast, Mg/Ca ratios show a statistically significant difference between spot 1 and spot 4 (Wilcoxon test, p < 0.05; Fig. 3c), indicating systematic changes in Mg incorporation across shell growth. Similarly, Li/Mg ratios differ significantly between spot 1 and spot 4 (Welch’s t-test, p < 0.05; Fig. 3d).

## Discussion

We evaluated for the first time the applicability of Li/Mg thermometry in *H. inflatus* shells, a widely used temperature proxy^14–16^, and show that Li/Mg ratios robustly reflect upper-water temperature conditions at sampling locations. Interpreting this relationship requires consideration of calcification timing: ecological evidence indicates that shell formation occurs over a restricted ~ 7–8 month interval^29^, with potentially variable growth across ontogeny. As a result, shell carbonate integrates environmental conditions over weeks to months, whereas in situ temperature measurements represent conditions at collection. Accordingly, the Li/Mg–temperature relationship reflects the prevailing upper-water temperature regime rather than instantaneous calcification temperatures. It should also be noted that this Li/Mg thermometer has not yet been independently tested on specimens with controlled or known growth conditions. While the correlation with δ1⁸O-derived temperatures is promising, future culture experiments and time-resolved field studies are needed to validate the robustness of this proxy across different life stages and environmental settings.

Apart from an early study investigating trace elemental incorporation in pteropods^10^, there has been no study looking at a suite of trace metals in pteropods since. However, several studies have investigated the relationship between environmental signals and trace elemental signature in shells of various other groups. Jurikova and colleagues^13^ examined Li/Ca and Mg/Ca ratios in modern brachiopod shells and reported a moderate variability in Li/Ca (34–39.1 μmol/mol) and Mg/Ca (5.79 to 7.39 mmol/mol). Another study assessed elemental ratios in the aragonitic gastropod *Turbo torquatus* from multiple locations in Western Australia^11^. Their results yielded an average Li/Ca ratio of 7.1 μmol/mol, but they noted that the Li concentrations were too variable to serve as reliable environmental indicators. Average Mg/Ca values were with ca. 0.4 mmol/mol markedly lower than in brachiopods and more similar to the values we measured on pteropods (0.2–0.8 mmol/mol, Table 1). Another study analyzed elemental composition on another gastropod species, *P. caerulea,* and reported even lower Li/Ca ratios of 0.0031–0.0052 μmol/mol, associated with low Mg/Ca values of 0.52–0.78 mmol/mol^12^. It is noteworthy that all three gastropods, *Turbo, Patella* (aragonite shell parts*)*, and *Heliconoides*, display similar Li/Ca and Mg/Ca values^11,12^ This suggests similar Li and Mg incorporation mechanisms across the Gastropoda. These similarities among mollusks seem to be mirrored in their suitability as proxy archives, e.g. *Heliconoides* (this study) and *Mytilus*^34^. It remains to be studied which fractionation processes underpin the mostly robust Li/Mg vs temperature relationships in gastropods and other calcifiers. An exception is the limpet *Patella ulgate* which shows no correlation between Li/Mg and temperature^35^. In the above-mentioned sister-species *P. caerulea*, Li/Mg is influenced by carbonate chemistry induced calcification rate changes^12^. If such a carbonate chemistry effect should be detectable in *P. ulgate* as well, this would suggest that the temperature effect on Li/Mg is not a calcification rate effect in disguise. Whatever the mechanism behind the Li/Mg-temperature relationship is, the latter seems to be widespread among marine calcifiers since it can be observed in corals and foraminifera as well^15^. Different groups of calcifiers differ considerably in their calcification mechanisms^36^ suggesting that the Li/Mg-temperature relationship is not linked to a particular group-specific mechanism. This inference is supported by the fact that two gastropods, *H. inflatus* (this study) and *P. vulgata*^35^ show different Li/Mg-temperature relationships although they belong to the same group of calcifiers.

**Fig. 4:**
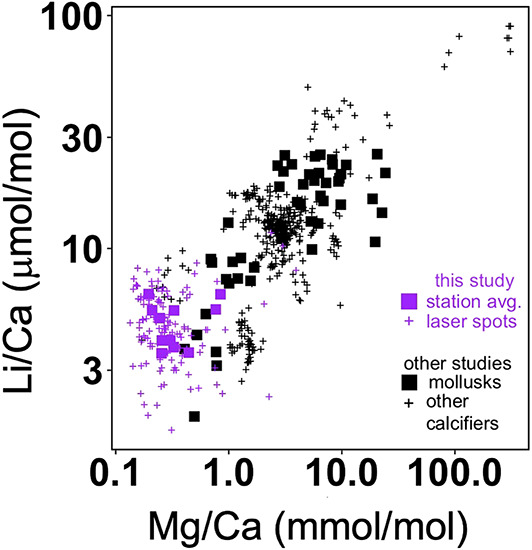
Relationship between Li/Ca (μmol/mol) and Mg/Ca (mmol/mol) across different groups of organisms. Purple indicates results from this study, with purple squares indicating average values (Table 1), purple crosses indicating laser spots (Table S1). Data from various published datasets are represented in black. Black squares indicate data from other mollusks^11,12,34,38,39^, crosses indicate data from corals^16,17,37,40,41^, planktic and benthic foraminifera^42–46^, red algae ^46^ and brachiopods^13,34,47,48^. Note logarithmic scales.

Marriott and coworkers^37^ compared Li/Ca ratios and lithium isotopic compositions in abiotic and foraminiferal CaCO₃, concluding that biological control is generally limited but not absent. They highlighted the importance of mineralogy, temperature, and salinity, while also recognizing the influence of species-specific physiological factors in modifying trace elemental uptake. A strong association between Mg and Li in biogenic carbonates was pointed out, suggesting similar transport mechanisms of these two elements in mollusks as identified by Dellinger an colleagues^34^. The Li/Ca and Mg/Ca values we report here fit well into their overall correlation of Li/Ca versus Mg/Ca^34^, aligning well with values from other marine calcifiers, including mollusks (Fig. 4). Our data from pteropods can be found on the lower end of the range of trace elemental/ calcium ratios along with values reported by Langer and colleagues^12^ for the gastropod *P. caerulea*.

While recent studies have advanced our understanding of pteropod shell ultrastructure^49^ the mechanisms of their biomineralization remain poorly understood. However, extensive research on other mollusks provides useful insights that may inform hypotheses about pteropod shell formation^36^. In mollusks, calcification was traditionally assumed to occur within the extrapallial fluid (EPF)—a semi-isolated compartment located between the shell and mantle epithelium. The EPF is generally assumed to be similar in ionic composition to seawater, but enriched with organic macromolecules such as proteins and polysaccharides that are believed to modulate crystal nucleation and growth^50–54^. The precipitation of shell calcium carbonate directly from the EPF, however, has become increasingly unlikely since multiple avenues of research have shown that the mantle epithelium most likely is in close contact with the shell surface at the site of crystallization^55–59^. Based on this idea, a distinction between two ion transport scenarios, the “EPF scenario” and the “mantle scenario” was proposed by Langer and colleagues^12^. The latter authors suggested that in the EPF scenario the fractionation of trace elements should be explicable in terms of inorganic precipitation from seawater, whereas in the mantle scenario no such simple explanation is expected because selective ion transport can, and likely will, lead to fractionation patterns markedly different from fractionation in inorganic precipitation experiments. Fractionation patterns can be analyzed from different perspectives.

A useful parameter to look at in this regard is the partitioning coefficient (K_D_) which is defined as the molar ratio of the trace element (TE) and Ca in calcium carbonate (here: aragonite) and (TE/Ca)_SW_ to that in the seawater (Eq. 4). The K_D_ not only reflects the chemical partitioning between seawater and biomineral but can also be influenced by biological ion transport, partial ion selectivity, and isotopic fractionation during calcification^60–63^, for which we refer the reader to Branson et al.^60^ for a detailed discussion. The K_D_ shows if the element is enriched (K_D_ > 1) or depleted (K_D_ < 1) in the biomineral relative to seawater. Assuming average Mg/Ca ratio of surface Atlantic seawater (~ upper 200 m) of approximately 5.1 mol/mol^64^, the calculated K_DMg_ for our pteropod values falls between 0.04–0.17 × 10^–3^ (Table S3), whereas inorganic K_DMg_ for aragonite of ca. 1–2 × 10^–3^ have been reported^65^.

Little information on seawater Li/Ca variability has been reported, so we assume a seawater Li/Ca ratio of 0.0025 mol/mol here^17^, resulting in a range of K_DLi_ for our Li/Ca values of ca. 1.4–2.5 × 10^–3^ (Table S3). In comparison, inorganic K_DLi_ for aragonite have been reported on the order of 3–4 × 10^–3^^37^. Interestingly, our average values for K_DMg_ and K_DLi_ (0.1 and 1.9 × 10^–3^, Table S3), align well with those observed in *P. caerulea,* another gastropod species (0.1 and 2.2 × 10^–3^)^12^. Our calculated partitioning coefficients (K_D_), namely 0.04–0.17 × 10^–3^ for K_DMg_ and 1.4–2.5 × 10^–3^ (Table S3) for K_DLi_ show that the K_DLi_ is explicable in the EPF scenario, since the K_DLi_ close to values reported for inorganically precipitated aragonite (3–4 × 10^–3^)^37^ whereas the KD_Mg_ is not (inorganic values: 1–2 × 10^–3^)^65^. The same holds for *P. caerulea*, where also the K_D_s of Sr, B, and U were inexplicable in the EPF scenario^12^. It would be helpful to analyze K_D_s of Sr, B, and U in pteropods as well to uncover how far similarities in absolute fractionation (K_D_) between mollusks go. At any rate, it can be tentatively concluded that pteropods do not calcify according to the EPF scenario, because for this to be so, the K_D_ of every trace element should be seawater-like.

Another similarity in the fractionation behavior of *H. inflatus* and *P. caerulea* is the apparent sensitivity of K_DLi_ to environmental and physiological parameters. In *P. caerulea*, K_DLi_ shows a negative correlation with calcification rate^12^. In *H. inflatus*, K_DLi_ exhibits a negative correlation with temperature (Fig. 2a), which may indirectly reflect changes in calcification rate, as temperature and calcification rate are generally positively correlated in pteropods (up to a thermal optimum)^66^. By contrast, inorganic precipitation experiments show a positive relationship between K_DLi_ and precipitation rate^67^. This is another indication that the mantle scenario is operative in pteropods, as it most likely is in other mollusks. The positive K_DMg_ rate dependence in *P. caerulea* and the insignificant temperature dependence in *H. inflatus* are the only difference in fractionation patterns between these two gastropods. It is clearly warranted to analyze more trace elements and more aspects of their fractionation patterns (such as dependence on seawater chemistry) in pteropods. These data would help to understand whether pteropods follow the general mollusk biomineralization mechanism.

Systematic intra-shell variability in TE/Ca within shells has been reported for other calcifiers, e.g. Fehrenbacher and colleagues documented ontogenetic trends in Mg/Ca values along foraminiferal chamber growth^68^. Here, we find an increase in Mg/Ca and concurrent decrease in Li/Mg from spot 1 (very early juvenile- veliger) to spot 4 (older shell material; Fig. 3), which may suggest systematic changes during shell growth. Applying the exponential (linear) Li/Mg–temperature calibration (Eq. 2) yields a reconstructed temperature of approximately 18.5 (19.1) °C for the median Li/Mg ratio of laser spot 1 (23.6 µmol/mmol), whereas median values from laser spot 4 (8.9 µmol/mmol) correspond to markedly higher temperatures of approximately 32.4 (29.6) °C. A key question is whether the Li/Mg ratios measured in the final laser spots correspond to temperatures comparable to in-situ values. Mean Li/Mg ratios from the outermost shell portions of each specimen (laser spots 3 or 4), representing the latest growth stage prior to collection, yield reconstructed temperatures that are broadly consistent with contemporaneous in-situ measurements at the sampling stations. Using the linear calibration (Eq. 1), reconstructed temperatures range from 18.9 to 31.0 °C, overlapping with—but slightly exceeding—the observed 50 m temperatures of 16.7–28.2 °C, when excluding the southernmost station at the edge of the calibration range. In addition to seasonal variability in ambient temperature, these ontogenetic differences in Li/Mg ratios may reflect changes in biomineralization processes during early shell formation, shifts in habitat depth occupied over the life cycle, or a combination of these factors.

Shells of *H. inflatus* have been reported to exhibit microstructural heterogeneity. Most of the shell consists of fibrous material arranged in a helical structure, corresponding to laser spots 1–3 (Fig. 3). In contrast, later ontogenetic shell material near the exterior, display a crossed-lamellar microstructure^49^. However, since the fully developed crossed-lamellar microstructure occurs only at the extreme outer shell edge, which is fragile and rarely preserved, laser spot 4 likely sampled predominantly late ontogenetic shell material composed of helical structure with possible contributions from partially developed crossed-lamellar layers. This structural transition occurs over a narrow spatial interval and therefore does not represent a discrete microstructural boundary but rather a gradual ontogenetic modification of shell architecture. Such ontogenetically controlled variations in shell microstructure and mineralization pathways during pteropod shell growth may influence trace-element incorporation and could therefore partly explain the differences in Li/Mg ratios between earlier-formed shell portions (laser spots 1–3) and later formed shell material (laser spot 4), independent of external environmental conditions. However, these microstructural differences cannot account for the variations observed within laser spots 1–3 themselves, where a distinct decreasing trend in Li/Mg ratios is already evident (Fig. 3d). Consequently, additional explanations must be considered.

*Heliconoides inflatus* exhibits brooding behaviour^69^, whereby embryos are retained within the mantle cavity until they are released as veligers (approximately 70 µm in diameter). If initial shell formation occurs while embryos or veligers are still brooded by the adult, the resulting Li/Mg signature may reflect the environmental conditions experienced by the brooding individual rather than those encountered by free-living early life stages. This process would primarily affect the earliest shell material (laser spot 1), providing a potential explanation for the difference in Li/Mg ratios—and consequently reconstructed temperatures—between embryonic shell portions and those formed during later ontogenetic stages. However, this mechanism alone cannot explain the progressive decrease in Li/Mg ratios observed from laser spots 2 to 4 (Fig. 3d).

Pteropods are not currently known to actively dissolve substantial portions of previously precipitated shell carbonate to create internal space, in contrast to some predatory gastropods such as Conidae^70^. Shell modification in pteropods is instead thought to occur primarily through external growth and episodic repair^71^ rather than extensive internal resorption. Consequently, previously formed carbonate is likely preserved within the shell structure, allowing earlier growth stages to remain detectable in spatially resolved geochemical measurements.

The most important external factor to consider is temperature. Although the timing and environmental conditions of egg release in *H. inflatus* remain poorly constrained, sediment trap observations^29^ and the intra-shell geochemical data presented here (Table S1; Fig. 3d) provide insight into aspects of its life cycle. Interpreting the data in terms of a discrete seasonal spawning period is challenging due to opposing seasons encountered along the transect, the generally weak seasonality of tropical and subtropical waters, and the widespread occurrence of veligers, juveniles, and adults throughout the study area (K. Peijnenburg, pers. obs.). These observations suggest that the life cycle of *H. inflatus* differs from that of higher-latitude pteropod species and is unlikely to be tightly coupled to strong seasonal environmental variability. Nevertheless, temperature remains the most plausible external control on Li/Mg incorporation.

The relatively low reconstructed temperatures in embryonic and juvenile shell portions likely reflect formation during cooler phases of the annual cycle, potentially amplified by ontogenetic processes or brooding. Our previous δ^1^⁸O study on the same specimens showed strong agreement with contemporaneous seawater conditions^27^. In addition, because bulk shell analyses are dominated by the most recently deposited—and therefore biggest and thickest—material, the carbonate composition of whole shells in bulk analyses largely reflects recent environmental conditions. The apparent contrast with intra-shell Li/Mg variability arises from differences in temporal resolution: δ^1^⁸O integrates carbonate formed over the main calcification period, whereas laser-ablation analyses resolve earlier ontogenetic shell domains. Thus, shells are temporally integrated at the whole-shell scale but internally heterogeneous at finer resolution.

Although intra-shell Li/Mg variability might be consistent with temperature changes experienced during ontogeny, its quantitative interpretation remains dependent on the station-averaged calibration. In addition to temperature, other environmental factors, such as salinity, carbonate chemistry, and local nutrient conditions, may influence Li/Mg incorporation in pteropod shells. We note that surface waters at our sampling sites may experience seasonal variability and influence from equatorial currents, which could reach amplitudes comparable to the Li/Mg calibration range. Across our transect, surface salinities varied by several practical salinity units (Fig. 1b), which could affect ionic strength and the transport of Li and Mg into the calcifying fluid. While the strong correlation with temperature suggests that thermal effects dominate (Table 2), non-thermal influences cannot be fully excluded. Investigating these effects in controlled culture experiments or in surface sediment samples spanning salinity gradients would help disentangle temperature signals from other environmental drivers, and further test the robustness and universality of the Li/Mg paleothermometer in pteropods. Given these uncertainties, intra-shell Li/Mg should currently be interpreted as indicators of relative changes in environmental or physiological conditions during growth rather than as precise seasonal temperatures. Independent time-resolved datasets, such as sediment-trap records or culture experiments, will be required to test whether Li/Mg variations directly track seasonal cycles and to further decode the life-history information archived within pteropod shells. Sediment traps are useful for constraining the timing of the last shell material deposited, which is important because pteropod calcification may not be linear. Environmental variability throughout the full calcification period can then be assessed via intra-shell analyses.

The geological record contains a wealth of information about past climate change events, as past ocean temperature and chemistry can be derived from fossil calcium carbonate shells^72,73^.

The oldest fossil pteropod is 72.1 Ma, but pteropods do not become regular components of sediments before 55 Ma^74^. The present study shows that the studied species here, *H. inflatus,* is nicely suited for paleo-reconstructions, as the Li/Mg thermometer works well in pteropod shells. Additionally, the fact that these proxies cannot be only measured simultaneously on a single pteropod shell but also along the whorl of a pteropod renders them promising new proxy carriers, as this offers the potential to assess seasonal variation within one specimen.

The material comprising a single pteropod shell may, in theory, be precipitated at different depths and over multiple seasons. This is consistent with reports indicating that the average lifespan of several pteropod species is approximately one year^23^. In the case of *H. inflatus*, the lifespan is likely shorter, on the order of ~ 7–9 months^24^. Because *H. inflatus* preferentially inhabits surface waters^24,26^, where environmental parameters influencing shell trace-element incorporation (e.g., temperature and salinity) vary seasonally, the resulting shell geochemistry may integrate signals from changing conditions throughout the year. Sediment-trap studies would therefore be well suited to assess the influence of seasonality on the trace-element composition of pteropod shells. In addition, culturing experiments could be used to investigate the effects of other environmental parameters, such as carbonate chemistry, on trace-element incorporation, analogous to approaches commonly employed in foraminiferal studies^75–78^.

Correlations between trace elemental composition of pteropod shells and upper ocean water temperature show that the Li/Mg paleothermometer may be applicable in pteropods with the following regression (assuming an exponential relationship between Li/Mg and temperature, from Eq. 2):

3 $$T = \frac{{ln\left( {\frac{85.94}{{\frac{Li}{{Mg}}}}} \right)}}{0.070} \pm \sqrt {\left( {4.63} \right)^{2} + \left( {2.86 ln\left( {\frac{85.94}{{\frac{Li}{{Mg}}}}} \right)} \right)^{2} }$$

where T is Temperature (°C) and Li/Mg is expressed in μmol/ mmol (p < 0.05, R^2^ = 0.73). Uncertainties in reconstructed temperatures can be obtained by propagation of the regression parameter errors and analytical uncertainty in Li/Mg following standard Gaussian error propagation.

Comparing the standard deviation of individual Li/Mg spots (≈4.0 µmol/mmol) with the slope of the exponential calibration (Eq. 2) provides a rough estimate of the resolvable temperature difference for station averages. For *H. inflatus*, this corresponds to ~ 1–2 °C for typical to high Li/Mg values (~ 0.37–35.3 µmol/mmol). This reflects sensitivity rather than full propagated uncertainty; including intra-shell variability and analytical reproducibility (RSD 23% for Li/Ca, 37% for Mg/Ca) would increase the range for most stations. Only stations with very low Li/Mg variability (< 1.3 µmol/mmol) approach this minimal resolvable difference. We note that the estimated 1–2 °C resolution refers to station-averaged Li/Mg values and the associated exponential calibration. In practice, *H. inflatus* shells likely grow over multiple depths and months, so the actual temperature signal recorded by an individual shell may integrate seasonal and vertical variability. Thus, while the calibration indicates potential sensitivity under idealized conditions, natural intra-shell and vertical heterogeneity will reduce the effective temporal resolution of reconstructed temperatures.

We emphasize that the calibration presented here is based on a single pteropod species and may not be directly transferable to other species. Differences in growth duration, depth habitat, and physiological regulation are likely to influence elemental incorporation, meaning that species-specific calibrations may ultimately be required. Consequently, the relationship reported here should be regarded as a first-order framework for Li/Mg-based temperature reconstruction rather than a universally applicable proxy across all pteropods. We note that resolving the effects of calcification timing is critical for interpreting Li/Mg signals, particularly when extending this approach to taxa with continuous annual growth.

*Heliconoides inflatus* is a pteropod species that has a global distribution in tropical and subtropical waters (including the Caribbean, Mediterranean and Indo-Pacific). Therefore, it is a good proxy carrier to assess surface water variations over paleo timescales worldwide. One limitation is the occurrence of well-preserved pteropod shells in sediments confined to waters above the lysocline of aragonite. However, there are plenty of sediment cores available in which *H. inflatus* is abundant, well preserved and where the calibrations reported here can be applied^28,30,31,79–81^.

## Methods

### Pteropod collection

Bulk zooplankton samples were collected during the Atlantic Meridional Transect Cruise 22 (AMT22), which took place from October 19 to November 16, 2012. Oblique bongo net tows (200 µm and 333 µm mesh sizes) were conducted from an average depth of 361 m up to the sea surface, primarily during the pre-dawn hour. Following collection, specimens were immediately preserved in 96–99% ethanol, with the ethanol replaced within 12–24 h. Samples were then stored at –20 °C until further analysis. Pteropods were sorted from these plankton samples at 11 stations spanning latitudes from 31°N to 38°S (see Table S1). We analyzed only specimen of *H. inflatus,* which were abundant throughout the transect. Stations 62 and 66 correspond to southern Atlantic occurrences of *Heliconoides inflatus*, identified as *H. inflatus* S following the biogeographic classification of Burridge et al.^82^.

### Seawater parameters: temperature and salinity

Seawater temperature and salinity in the upper 500 m of the water column were obtained by conductivity-temperature-depth (CTD) casts (Sea-Bird Electronics, models: ocean logger, SBE45, 9plus). Sensors were calibrated and data archived by the British Oceanographic Data Centre (BODC). Furthermore data from the World Ocean Database (WOD)^32^ were used to generate surface distribution maps of the Atlantic for temperature and salinity (Fig. 1). Plots present average values from October through November in order to obtain a representation of the typical surface distribution of these parameters during the period of the cruise (10/13/2012 to 11/19/2012). The WOD data collection contained all surface data available from 1986 to 2011. In-situ temperatures used for calibration are interpreted as representative habitat temperatures characteristic of each station rather than instantaneous calcification temperatures. Throughout this study, “temperature” refers specifically to the mean environmental temperature representative of the upper water column habitat occupied by pteropods during calcification, derived from station-averaged hydrographic observations (0–50 m depth interval unless otherwise specified). When alternative temperature metrics are discussed (e.g., surface temperature or instantaneous sampling temperature), these are explicitly stated.

## LA-ICP-MS analysis

### Sample preparation for LA-ICP-MS

Specimens were picked from the plankton samples and rinsed in 3 × ultrapure water and dried in Krantz slides. No oxidative cleaning was applied prior to LA-ICP-MS analysis. Chemical cleaning procedures can alter or partially dissolve delicate aragonitic pteropod shells and potentially modify trace-element distributions at the microscale. Instead, contamination was assessed directly using time-resolved ablation profiles and element screening (see below), allowing exclusion of contaminated signal intervals while preserving primary shell chemistry.

Specimens were transferred onto a double-sided carbon tape mounted to a slide.

### Laser ablation protocol

The LA-ICP-MS (laser ablation-inductively coupled plasma-mass spectrometry) system at Kiel University was used to determine elemental concentrations of pteropod shells. The LA-ICP-MS system consisted of a quadrupole mass spectrometer (Agilent 7500 s) connected to a 193 nm excimer laser ablation system (Coherent GeoLas HD), equipped with a Zurich-type two-volume ablation cell. Helium (~ 1L/min) was used as the carrier gas with 14 mL/min H_2_ added to increase sensitivity. The pulse repetition rate was set to 6 Hz, and energy density was ~ 3 J cm^-2^ while using an ablation beam diameter of 60 μm. Ion counts of ^7^Li, ^24^ Mg, ^27^Al, ^43^Ca ^44^Ca, ^57^Fe were used to calculate elemental concentrations. A complete measurement cycle of the mass spectrometer through all masses took 0.5 s. The ablation profiles were checked for potential surface contaminations using ^27^Al and ^57^Fe. A glass reference material (SRM NIST 610)^83^ was ablated three times and a pressed-powder nanopellet^84^ of JCt-1 (aragonitic giant clam *Tridacna gigas*)^85^ was used as a consistency standard between every 10 samples. The glass standard was ablated at a higher energy density (~ 13 J cm^-2^). Assuming 40wt% (m/m) calcium in calcite, ^43^Ca was chosen as an internal standard, while counts for ^44^Ca were used to check for consistency.

### Laser data processing

Using the software Glitter (software for LA data reduction)^86^, data reduction followed standard laser ablation ICP-MS protocols (e.g., Longerich et al., 1996^87^), including inspection and removal of outliers, signal smoothing, and correction for instrumental drift by bracketing samples with repeated analyses of NIST SRM 610 standard. Background intensities were determined from pre-ablation intervals and subtracted from each data point. Only stable portions of the ablation signal corresponding to penetration through the shell wall were integrated. Initial surface signal and final low-count intervals were excluded. Potential contamination was evaluated using elevated ^27^Al and ^57^Fe signals. Integration intervals were manually selected to exclude sections showing enrichment in these elements relative to Ca. Mean TE/Ca values for each profile were calculated by normalizing measured intensities to the known trace element concentrations of the drift-corrected NIST SRM standard^83^, using ^43^Ca as an internal standard. Shell wall TE/Ca averages were obtained by integrating TE/Ca ratios across the ablation profile, excluding elevated TE/Ca values commonly observed at the start of ablation (see Fig. S3). During the measurements, the laser ablated sequentially deeper parts of the test, and measurements were stopped once the laser had fully protruded through the test. Since the focus changes during ablation, this means that the exact end point was hard to determine precisely during measurements, and we usually kept ablating for a bit longer. When analyzing the laser profiles the exact end point can be easily determined, as the Ca signal tends to decreases sharply, once the laser has protruded through the shell for the first time. Each laser ablation analysis consisted of a pre-ablation background acquisition of 20 s followed by 40–60 s of signal acquisition. The first ca. 5–10 s of ablation was excluded to avoid potential surface contamination (identified via e.g. Al. peaks), and elemental ratios were calculated from the subsequent stable signal interval (typically 10–40 s), identified by constant Ca count rates and absence of transient spikes. Several standards were repeatedly analyzed throughout the 2 days of data collection period (Table S4): NIST SRM glass 610 (n = 29), and pressed powder pellets of JCT1 (n = 12), B393 (n = 18) and RS3 (n = 9). The RSD of these repeated measurements of Li/Ca and Mg/Ca ratio ranges from 2.2% for (Li/Ca and Mg/Ca) on the N610 glass to a maximum of 24.1% (Li/Ca) and 4.1% (Mg/Ca) of the pressed powder tablet of JCt1.

### Calculating average TE/Ca ratios and KDs

Three individual specimens were analyzed per station, with three to four laser spot measurements conducted per shell, yielding a total of 104 single-spot measurements on pteropod shells (Tab S1). All reported averages are unweighted, implicitly assuming that each analyzed shell region contributes equally to the overall calcium carbonate budget of the shell. This approach was adopted in the absence of detailed quantitative information on shell thickness and mass distribution across shell ontogeny. Station-level means were calculated from the three specimen averages. The standard error (SE) for each station was calculated from the standard deviation of the three specimen averages divided by the square root of the number of specimens (n = 3 or 4). Single spot measurements from different specimens were averaged per station.

Using reported seawater Mg/Ca (5.1 mol/mol)^64^ and Li/Ca (0.0025 mol/mol)^17^, the empirical partition coefficient for these elements in pteropod shells (K_D_) was calculated according to:

4 $$K_{D} = \frac{{\left( {\frac{TE}{{Ca}}} \right)_{CC} }}{{\left( {\frac{TE}{{Ca}}} \right)_{SW} }}$$

where (TE/Ca)_CC_ refers to the measured molar ratio of the trace element (TE) and Ca in calcium carbonate (here: aragonite) and (TE/Ca)_SW_ to that in the seawater.

Throughout this study, ‘individual’ refers to a single pteropod specimen. Spot-level measurements within each specimen are explicitly referred to as ‘spots’. All reported K_D_ values represent averages across stations (Tab S3).

### Statistical analyses

Outlier screening was limited to analytical quality criteria (signal stability, detection limits, and contamination checks), rather than geochemical thresholds, to avoid subjective data exclusion in the absence of established baseline variability for pteropods.

Regression analyses (linear and exponential) were performed to analyze correlation between station-averaged pteropod trace elemental ratios (namely Li/Ca, Mg/Ca, Li/Mg) and environmental parameters (temperature, salinity) at several depths (2, 25, 50, 75, 100, 200, 250, 300 m). Furthermore, we performed regressions against d^18^O, as this has been found on the same set of plankton samples to be a good temperature proxy^27^. Data was tested for normality using Shapiro test, normal distributed data was compared using Welch’s t-test, if not, Wilcoxon test was performed. All analyses were performed in R ( version 4.4.1^88^).

## Supplementary Information

Below is the link to the electronic supplementary material.

Supplementary Material 1.

## Data Availability

All Li/Mg, Mg/Ca, and Li/Ca ratios measured in this study are provided in the Supplementary Information. Shell stable carbon and oxygen isotope data can be found in our previous publication: Keul, N. et al., Scientific Reports 7, 12,645 (2017). Any additional information or analysis scripts are available from the corresponding author upon reasonable request.
