# Supplementary material for: A new Li/Mg paleothermometer from pteropod shells
Source: Sci Rep. 2026 Jun 11;16:18190. doi: 10.1038/s41598-026-55990-z (PMC13261124; doi:10.1038/s41598-026-55990-z)
Supplement: Supplementary file 1 — Supplementary Material 1. [file 41598_2026_55990_MOESM1_ESM.docx]

**Supplementary Information for:**

**A New Li/Mg Paleothermometer from Pteropod Shells**

N. Keul^1^, D. Garbe-Schönberg^1^, V. Kitidis^2^, G. Langer^3^, K.T.C.A. Peijnenburg^4,5^

^1^ *Institute of Geosciences*, *Christian-Albrechts-Universität zu Kiel, Ludewig-Meyn-Str.10,*

*24118 Kiel, Germany*

^2^ *Plymouth Marine Laboratory, Plymouth, United Kingdom*

^3^ *Institute of Environmental Science and Technology (ICTA-UAB), Universitat Autònoma de Barcelona, 08193, Bellaterra, Spain*

^4^ *Naturalis Biodiversity Center, P.O. Box 9517, 2300 RA Leiden, The Netherlands*

^5^ *Institute for Biodiversity and Ecosystem Dynamics (IBED), University of Amsterdam, P.O. Box 94248, 1090 GE Amsterdam, The Netherlands*

**Table of contents:**

Figure S1. Distribution plots of Li/Ca, Mg/Ca, Li/Mg and Temperature versus Latitude

Figure S2. Relationship between Li/Mg and d^18^O_pteropod_

Figure S3. Exemplary, laser ablation profiles of randomly selected laser spots

Figure S4. Representative screenshots obtained during laser ablation of pteropod shells.

Table S1: Trace elemental composition of individual laser spots

Table S2. Statistical parameters for linear correlation between average pteropod shell Li/Ca

and Mg/Ca and temperature and salinity

Table S3: Partitioning coefficients for Li (K_DLi_) and Mg (K_DMg_)

**
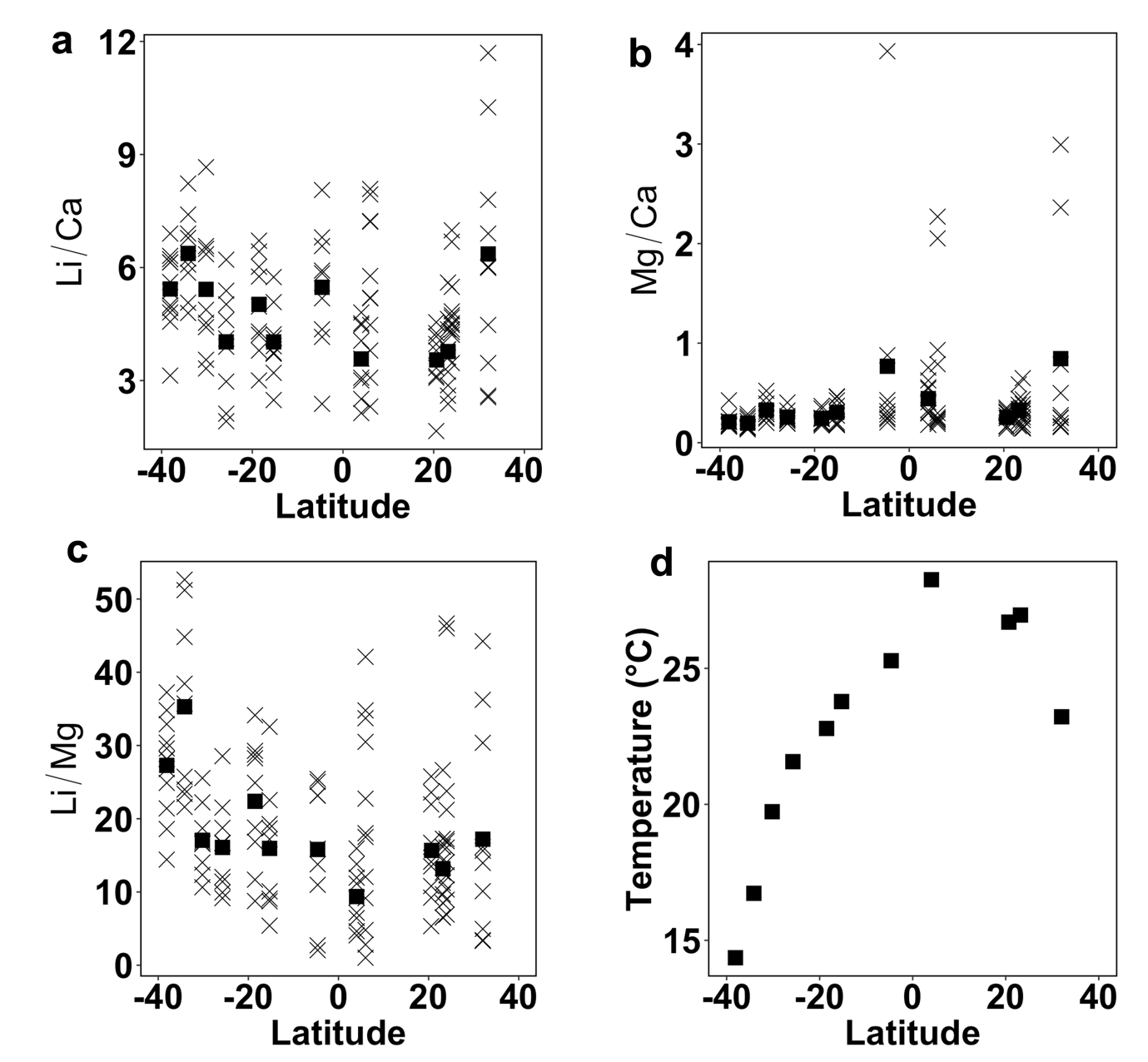
**

**Figure S1.** Latitudinal variation of pteropod trace-element ratios and surface-ocean parameters. (**a**) Li/Ca (μmol/mol), (**b**) Mg/Ca (mmol/mol), (**c**) Li/Mg (μmol/mmol) and (**d**) Temperature at 50m depth versus latitude in the Atlantic Ocean. Crosses depict individual measurements; Station averages are indicated by closed squares. Negative latitude denotes the southern hemisphere. Data can be found in Tab. 1.


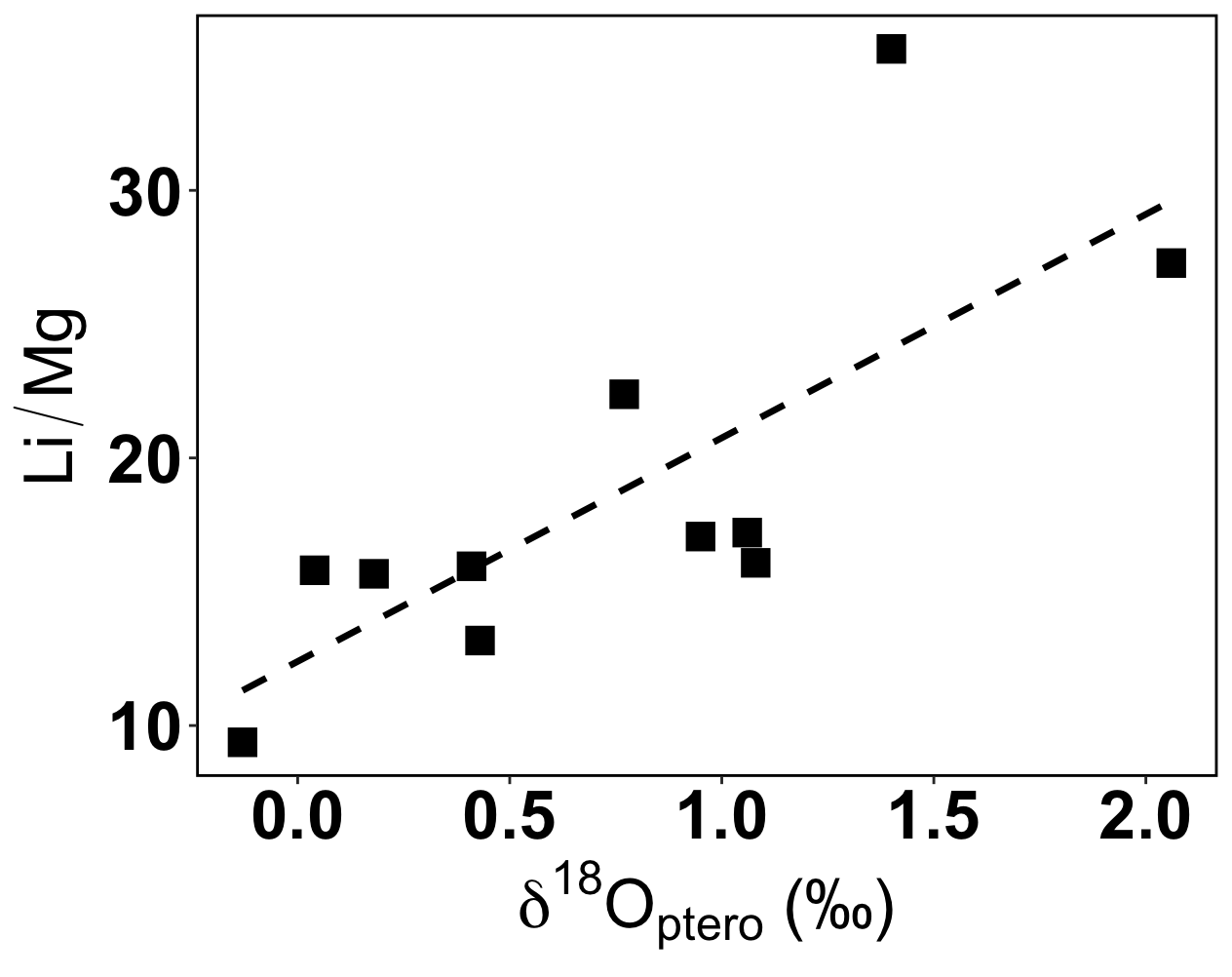


Figure S2. Relationship between Li/Mg (μmol/mmol, this study) and δ^18^O_pteropod_ (Keul et al., 2017). Dashed lines depict statistically significant (p<0.05) linear regressions.


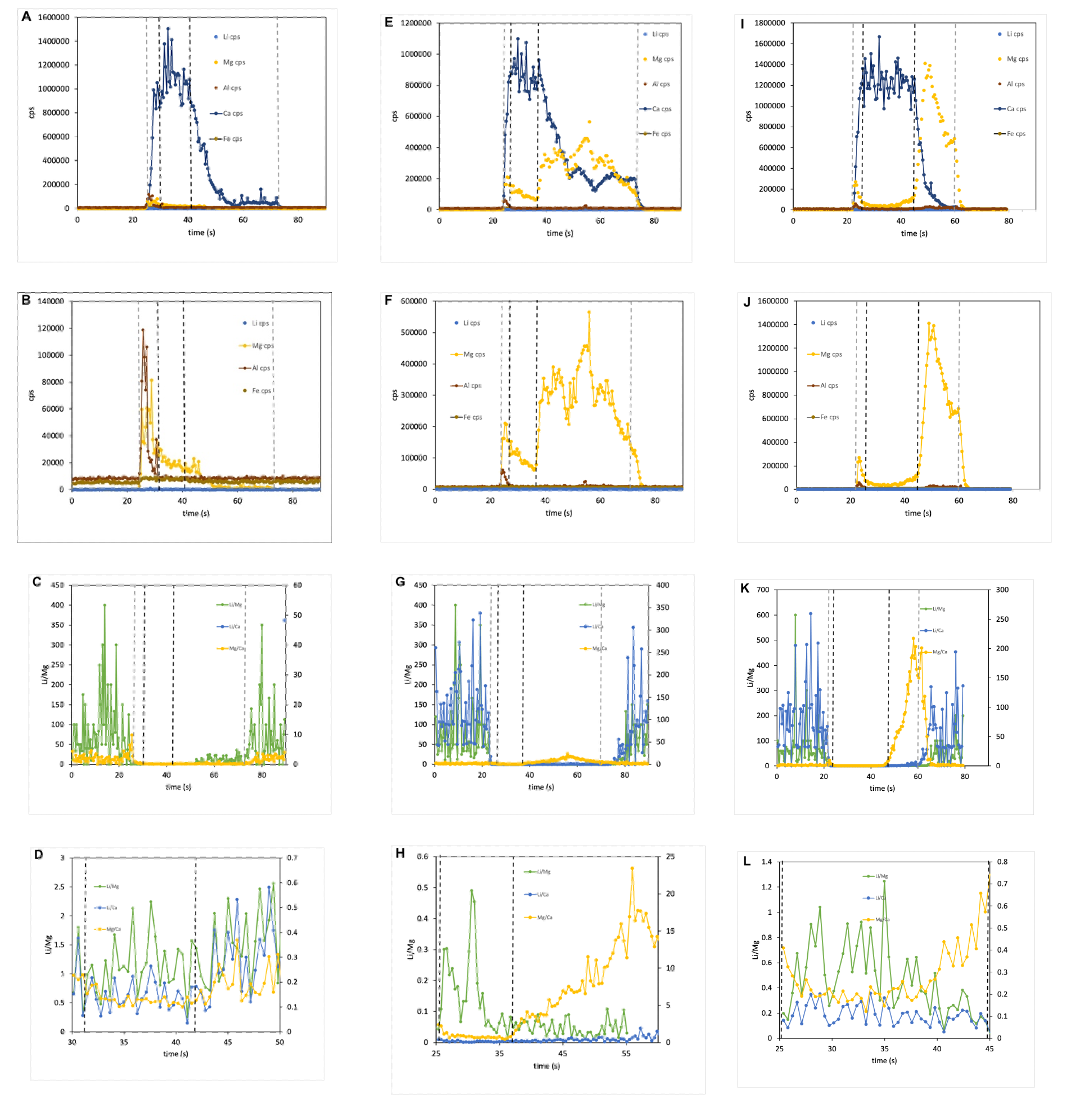


Figure S3. Exemplary, laser ablation profiles of randomly selected laser spots. Grey vertical lines indicate start and stop of laser ablation, black lines indicate integrated profile. Please note all profiles are displayed from raw data (in counts per second, cps).

**a-d)** Pteropod ID 3722151A **a)** cps versus time in seconds (s) of Ca; **b)** cps of Al, Mg and Fe; **c)** ratios of Li/Ca *10^5^ (secondary y-axis; cps/cps), Mg/Ca *10^2^ (secondary y-axis; cps/cps) and Li/Mg (primary z-axis; cps/cps) and **d)** same as c), but zoomed into integrated time window.

**e-h)** Pteropod ID 4022151D **e)** counts per second (cps) versus time in seconds (s) of Ca; **f)** cps of Al, Mg and Fe; **g)** ratios of Li/Ca *10^5^ (secondary y-axis; cps/cps), Mg/Ca *10^2^ (secondary y-axis; cps/cps) and Li/Mg (primary z-axis; cps/cps) and **g)** same as g), but zoomed into integrated time window.

**i-l)** Pteropod ID 1822232B **i)** counts per second (cps) versus time in seconds (s) of Ca; **j)** cps of Al, Mg and Fe; **k)** ratios of Li/Ca *10^5^ (secondary y-axis; cps/cps), Mg/Ca *10^2^ (secondary y-axis; cps/cps) and Li/Mg (primary z-axis; cps/cps) and **l)** same as k), but zoomed into integrated time window.


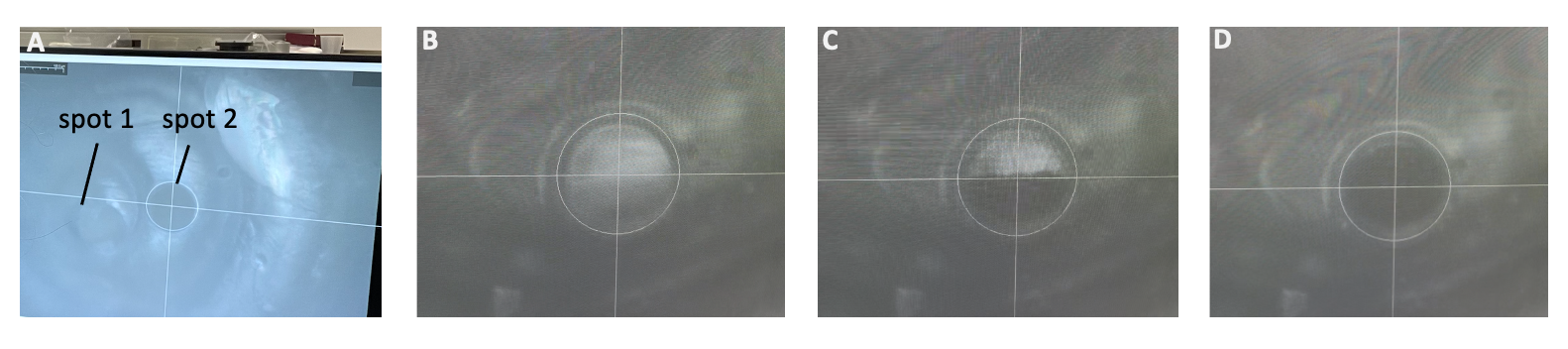


Figure S4. Representative screenshots obtained during laser ablation of pteropod shells. **A)** Location of laser spots 1 and 2. **B–D)** Sequence illustrating the ablation process before, during, and after penetration of the shell wall. Beam diameter is 60μm.

| pteropodID  (internal) | cruise | Latitude | spot  number | Li/Ca | Mg/Ca | Li/Mg |
| --- | --- | --- | --- | --- | --- | --- |
| 3722151A* | AMT22 | 32.02 | 1 | 6.02 | 0.17 | 36.24 |
| 3822151B | AMT22 | 32.02 | 2 | 6.90 | 0.16 | 44.24 |
| 3922151C | AMT22 | 32.02 | 3 | 2.56 | 0.25 | 10.11 |
| 4022151D* | AMT22 | 32.02 | 4 | 2.60 | 0.78 | 3.31 |
| **22151** | **pteropod avg.** | **32.02** |  | **4.52** | **0.34** | **23.47** |
| 4122152A | AMT22 | 32.02 | 1 | 5.99 | 0.20 | 30.37 |
| 4222152B | AMT22 | 32.02 | 2 | 4.48 | 0.28 | 16.14 |
| 4322152C | AMT22 | 32.02 | 3 | 3.46 | 0.25 | 13.96 |
| **22152** | **pteropod avg.** | **32.02** |  | **4.64** | **0.24** | **20.16** |
| 4422153A | AMT22 | 32.02 | 1 | 11.69 | 2.36 | 4.95 |
| 4422153B | AMT22 | 32.02 | 2 | 7.80 | 0.50 | 15.62 |
| 4522153C | AMT22 | 32.02 | 3 | 10.25 | 2.99 | 3.43 |
| **22153** | **pteropod avg.** | **32.02** |  | **9.91** | **1.95** | **8.00** |
| **2215** | **avg. station** | **32.02** |  | **6.36** | **0.84** | **17.21** |
|  | SE |  |  | 1.78 | 0.55 | 4.70 |
|  |  |  |  |  |  |  |
| 1322231A | AMT22 | 23.16 | 1 | 2.38 | 0.17 | 14.30 |
| 1422231B | AMT22 | 23.16 | 2 | 3.83 | 0.34 | 11.24 |
| 1522231C | AMT22 | 23.16 | 3 | 3.23 | 0.38 | 8.50 |
| 1622231D | AMT22 | 23.16 | 4 | 2.60 | 0.27 | 9.69 |
| **22231** | **pteropod avg.** | **23.16** |  | **3.01** | **0.29** | **10.93** |
| 1722232A | AMT22 | 23.16 | 1 | 4.36 | 0.27 | 16.17 |
| 1822232B* | AMT22 | 23.16 | 2 | 3.61 | 0.29 | 12.66 |
| 1922232C | AMT22 | 23.16 | 3 | 2.87 | 0.44 | 6.51 |
| **22232** | **pteropod avg.** | **23.16** |  | **3.61** | **0.33** | **11.78** |
| 2022233A | AMT22 | 23.16 | 1 | 5.60 | 0.21 | 26.64 |
| 2122233B | AMT22 | 23.16 | 2 | 4.63 | 0.27 | 17.27 |
| 2222233C | AMT22 | 23.16 | 3 | 3.83 | 0.59 | 6.53 |
| **22233** | **pteropod avg.** | **23.16** |  | **4.69** | **0.35** | **16.81** |
| **2223** | **avg. station** | **23.16** |  | **3.77** | **0.33** | **13.18** |
|  | SE |  |  | 0.49 | 0.02 | 1.84 |
|  |  |  |  |  |  |  |
| 2322251A | AMT22 | 20.57 | 1 | 3.12 | 0.14 | 21.96 |
| 2422251B | AMT22 | 20.57 | 2 | 3.80 | 0.35 | 10.81 |
| 2522251C | AMT22 | 20.57 | 3 | 4.26 | 0.30 | 14.12 |
| **22251** | **pteropod avg.** | **20.57** |  | **3.72** | **0.26** | **15.63** |
| 2622252A | AMT22 | 20.57 | 1 | 4.17 | 0.16 | 25.77 |
| 2722252B | AMT22 | 20.57 | 2 | 3.34 | 0.25 | 13.50 |
| 2822252C | AMT22 | 20.57 | 3 | 3.08 | 0.33 | 9.23 |
| **22252** | **pteropod avg.** | **20.57** |  | **3.53** | **0.25** | **16.17** |
| 2922253A | AMT22 | 20.57 | 1 | 3.97 | 0.17 | 23.59 |
| 3022253B | AMT22 | 20.57 | 2 | 4.55 | 0.27 | 16.72 |
| 3122253C | AMT22 | 20.57 | 3 | 1.66 | 0.31 | 5.33 |
| **22253** | **pteropod avg.** | **20.57** |  | **3.39** | **0.25** | **15.21** |
| **2225** | **avg. station** | **20.57** |  | **3.55** | **0.25** | **15.67** |
|  | SE |  |  | 0.10 | 0.01 | 0.28 |
|  |  |  |  |  |  |  |
| 3822371A | AMT22 | 4.03 | 1 | 3.00 | 0.33 | 9.21 |
| 3922371B | AMT22 | 4.03 | 2 | 4.48 | 0.63 | 7.16 |
| 4022371C | AMT22 | 4.03 | 3 | 2.51 | 0.54 | 4.64 |
| **22371** | **pteropod avg.** | **4.03** |  | **3.33** | **0.50** | **7.00** |
| 4122372A | AMT22 | 4.03 | 1 | 4.06 | 0.29 | 13.81 |
| 4222372B | AMT22 | 4.03 | 2 | 3.58 | 0.56 | 6.44 |
| 4322372C | AMT22 | 4.03 | 3 | 3.08 | 0.75 | 4.08 |
| **22372** | **pteropod avg.** | **4.03** |  | **3.57** | **0.53** | **8.11** |
| 4422373A | AMT22 | 4.03 | 1 | 2.14 | 0.18 | 11.96 |
| 4522373B | AMT22 | 4.03 | 2 | 4.81 | 0.30 | 15.94 |
| 4622373C | AMT22 | 4.03 | 3 | 4.52 | 0.41 | 11.13 |
| **22373** | **pteropod avg.** | **4.03** |  | **3.82** | **0.30** | **13.01** |
| **2237** | **avg. station** | **4.03** |  | **3.57** | **0.44** | **9.37** |
|  | SE |  |  | 0.14 | 0.07 | 1.85 |
|  |  |  |  |  |  |  |
| 4722431A | AMT22 | -4.62 | 1 | 4.36 | 0.32 | 13.78 |
| 4822431B | AMT22 | -4.62 | 2 | 6.80 | 0.43 | 15.91 |
| 4922431C | AMT22 | -4.62 | 3 | 5.85 | 0.25 | 23.16 |
| **22431** | **pteropod avg.** | **-4.62** |  | **5.67** | **0.33** | **17.62** |
| 5022432A | AMT22 | -4.62 | 1 | 5.92 | 0.24 | 25.05 |
| 5122432B | AMT22 | -4.62 | 2 | 4.16 | 0.38 | 10.98 |
| 5222432C | AMT22 | -4.62 | 3 | 8.06 | 3.93 | 2.05 |
| **22432** | **pteropod avg.** | **-4.62** |  | **6.04** | **1.52** | **12.69** |
| 5322431A | AMT22 | -4.62 | 1 | 5.18 | 0.20 | 25.43 |
| 5422431B | AMT22 | -4.62 | 2 | 6.57 | 0.28 | 23.16 |
| 5522431C | AMT22 | -4.62 | 3 | 2.38 | 0.88 | 2.71 |
| **22431** | **pteropod avg.** | **-4.62** |  | **4.71** | **0.46** | **17.10** |
| **2243** | **avg. station** | **-4.62** |  | **5.47** | **0.77** | **15.80** |
|  | SE |  |  | 0.40 | 0.38 | 1.56 |
|  |  |  |  |  |  |  |
| 5622491A | AMT22 | -15.29 | 1 | 3.21 | 0.19 | 17.20 |
| 5722491B | AMT22 | -15.29 | 2 | 4.23 | 0.47 | 9.08 |
| 5822491C | AMT22 | -15.29 | 3 | 2.48 | 0.46 | 5.40 |
| **22491** | **pteropod avg.** | **-15.29** |  | **3.31** | **0.37** | **10.56** |
| 6522492A | AMT22 | -15.29 | 1 | 4.13 | 0.18 | 22.54 |
| 6622492B | AMT22 | -15.29 | 2 | 3.74 | 0.37 | 10.09 |
| 6722492C | AMT22 | -15.29 | 3 | 3.71 | 0.42 | 8.73 |
| **22492** | **pteropod avg.** | **-15.29** |  | **3.86** | **0.33** | **13.79** |
| 6822493A | AMT22 | -15.29 | 1 | 5.75 | 0.18 | 32.55 |
| 6922493B | AMT22 | -15.29 | 2 | 5.08 | 0.27 | 18.69 |
| 7022493C | AMT22 | -15.29 | 3 | 3.91 | 0.20 | 19.28 |
| **22493** | **pteropod avg.** | **-15.29** |  | **4.91** | **0.22** | **23.51** |
| **2249** | **avg. station** | **-15.29** |  | **4.03** | **0.30** | **15.95** |
|  | SE |  |  | 0.47 | 0.05 | 3.89 |
|  |  |  |  |  |  |  |
| 7122511A | AMT22 | -18.52 | 1 | 3.80 | 0.20 | 18.79 |
| 7222511B | AMT22 | -18.52 | 2 | 6.42 | 0.19 | 34.13 |
| 7322511C | AMT22 | -18.52 | 3 | 4.30 | 0.37 | 11.68 |
| **22511** | **pteropod avg.** | **-18.52** |  | **4.84** | **0.25** | **21.54** |
| 7422512A | AMT22 | -18.52 | 1 | 4.97 | 0.17 | 28.75 |
| 7522512B | AMT22 | -18.52 | 2 | 5.76 | 0.20 | 29.27 |
| 7622512C | AMT22 | -18.52 | 3 | 4.22 | 0.25 | 16.86 |
| **22512** | **pteropod avg.** | **-18.52** |  | **4.98** | **0.21** | **24.96** |
| 7722513A | AMT22 | -18.52 | 1 | 6.02 | 0.24 | 24.91 |
| 7822513B | AMT22 | -18.52 | 2 | 6.71 | 0.24 | 28.26 |
| 7922513C | AMT22 | -18.52 | 3 | 3.00 | 0.34 | 8.76 |
| **22513** | **pteropod avg.** | **-18.52** |  | **5.25** | **0.27** | **20.64** |
| **2251** | **avg. station** | **-18.52** |  | **5.02** | **0.24** | **22.38** |
|  | SE |  |  | 0.12 | 0.02 | 1.32 |
|  |  |  |  |  |  |  |
| 8022571A | AMT22 | -25.75 | 1 | 2.12 | 0.23 | 9.15 |
| 8122571B | AMT22 | -25.75 | 2 | 4.61 | 0.40 | 11.41 |
| 8222571C | AMT22 | -25.75 | 3 | 5.02 | 0.27 | 18.75 |
| **22571** | **pteropod avg.** | **-25.75** |  | **3.92** | **0.30** | **13.10** |
| 8322572A | AMT22 | -25.75 | 1 | 1.92 | 0.19 | 9.94 |
| 8422572B | AMT22 | -25.75 | 2 | 5.39 | 0.32 | 16.71 |
| 8522572C | AMT22 | -25.75 | 3 | 2.97 | 0.25 | 12.04 |
| **22572** | **pteropod avg.** | **-25.75** |  | **3.43** | **0.25** | **12.90** |
| 0422573A | AMT22 | -25.75 | 1 | 4.11 | 0.19 | 21.52 |
| 0522573B | AMT22 | -25.75 | 2 | 6.21 | 0.22 | 28.54 |
| 0622573C | AMT22 | -25.75 | 3 | 3.90 | 0.23 | 16.65 |
| **22573** | **pteropod avg.** | **-25.75** |  | **4.74** | **0.21** | **22.24** |
| **2257** | **avg. station** | **-25.75** |  | **4.03** | **0.26** | **16.08** |
|  | SE |  |  | 0.38 | 0.03 | 3.08 |
|  |  |  |  |  |  |  |
| 0722601A | AMT22 | -30.2 | 1 | 6.57 | 0.26 | 25.55 |
| 0822601B | AMT22 | -30.2 | 2 | 4.42 | 0.20 | 22.23 |
| 0922601C | AMT22 | -30.2 | 3 | 6.50 | 0.39 | 16.56 |
| **22601** | **pteropod avg.** | **-30.2** |  | **5.83** | **0.28** | **21.45** |
| 1022602A | AMT22 | -30.2 | 1 | 3.55 | 0.29 | 12.34 |
| 1122602B | AMT22 | -30.2 | 2 | 4.88 | 0.29 | 16.88 |
| 1222602C | AMT22 | -30.2 | 3 | 6.35 | 0.45 | 14.03 |
| **22602** | **pteropod avg.** | **-30.2** |  | **4.93** | **0.34** | **14.42** |
| 1322603A | AMT22 | -30.2 | 1 | 3.32 | 0.31 | 10.64 |
| 1422603B | AMT22 | -30.2 | 2 | 4.53 | 0.24 | 18.71 |
| 1522603C | AMT22 | -30.2 | 3 | 8.66 | 0.52 | 16.62 |
| **22603** | **pteropod avg.** | **-30.2** |  | **5.51** | **0.36** | **15.32** |
| **2260** | **avg. station** | **-30.2** |  | **5.42** | **0.33** | **17.06** |
|  | SE |  |  | 0.26 | 0.02 | 2.21 |
|  |  |  |  |  |  |  |
| 1622621A | AMT22 | -34.15 | 1 | 4.79 | 0.13 | 35.73 |
| 1722621B | AMT22 | -34.15 | 2 | 5.07 | 0.22 | 23.40 |
| 1822621C | AMT22 | -34.15 | 3 | 5.86 | 0.24 | 23.99 |
| **22621** | **pteropod avg.** | **-34.15** |  | **5.24** | **0.20** | **27.71** |
| 1922622A | AMT22 | -34.15 | 1 | 6.90 | 0.15 | 44.82 |
| 2022622B | AMT22 | -34.15 | 2 | 8.23 | 0.16 | 52.64 |
| 2122622C | AMT22 | -34.15 | 3 | 6.79 | 0.26 | 25.73 |
| **22622** | **pteropod avg.** | **-34.15** |  | **7.31** | **0.19** | **41.06** |
| 2222623A | AMT22 | -34.15 | 1 | 6.08 | 0.16 | 38.44 |
| 2322623B | AMT22 | -34.15 | 2 | 7.41 | 0.14 | 51.21 |
| 2422623C | AMT22 | -34.15 | 3 | 6.24 | 0.29 | 21.61 |
| **22623** | **pteropod avg.** | **-34.15** |  | **6.57** | **0.20** | **37.09** |
| **2262** | **avg. station** | **-34.15** |  | **6.37** | **0.20** | **35.29** |
|  | SE |  |  | 0.60 | 0.00 | 3.96 |
|  |  |  |  |  |  |  |
| 3122661A | AMT22 | -38.11 | 1 | 4.56 | 0.16 | 28.13 |
| 3222661B | AMT22 | -38.11 | 2 | 6.14 | 0.18 | 34.82 |
| 3322661C | AMT22 | -38.11 | 3 | 4.81 | 0.16 | 29.61 |
| 3422661D | AMT22 | -38.11 | 4 | 5.62 | 0.26 | 21.41 |
| **22661** | **pteropod avg.** | **-38.11** |  | **5.28** | **0.19** | **28.49** |
| 3522662A | AMT22 | -38.11 | 1 | 3.13 | 0.17 | 18.60 |
| 3622662B | AMT22 | -38.11 | 2 | 5.00 | 0.19 | 26.78 |
| 3722662C | AMT22 | -38.11 | 3 | 6.31 | 0.21 | 30.38 |
| 3822662D | AMT22 | -38.11 | 4 | 6.90 | 0.21 | 32.92 |
| **22662** | **pteropod avg.** | **-38.11** |  | **5.33** | **0.19** | **27.17** |
| 3922663A | AMT22 | -38.11 | 1 | 6.22 | 0.17 | 37.26 |
| 4022663B | AMT22 | -38.11 | 2 | 4.92 | 0.17 | 28.18 |
| 4122663C | AMT22 | -38.11 | 3 | 5.37 | 0.22 | 24.89 |
| 4222663D | AMT22 | -38.11 | 4 | 6.14 | 0.43 | 14.42 |
| **22663** | **pteropod avg.** | **-38.11** |  | **5.66** | **0.25** | **26.19** |
| **2266** | **avg. station** | **-38.11** |  | **5.43** | **0.21** | **27.28** |
|  | SE |  |  | 0.12 | 0.02 | 0.67 |

**Table S1:** **Trace elemental composition of individual laser spots.** Columns contain: pteropod ID (for internal reference purposes), Station location information (cruise, Latitude), spot number on pteropod, trace elemental composition (Li/Ca in μmol/mol, Mg/Ca in mmol/mol, Li/Mg in μmol/ mmol). Average values for individual pteropods (bold), and station averages (bold, highlighted in purple), as well as standard error (SE, highlighted in purple) are calculated as well (SE). *next to pteropod ID indicates individual laser profiles in Fig. S3.


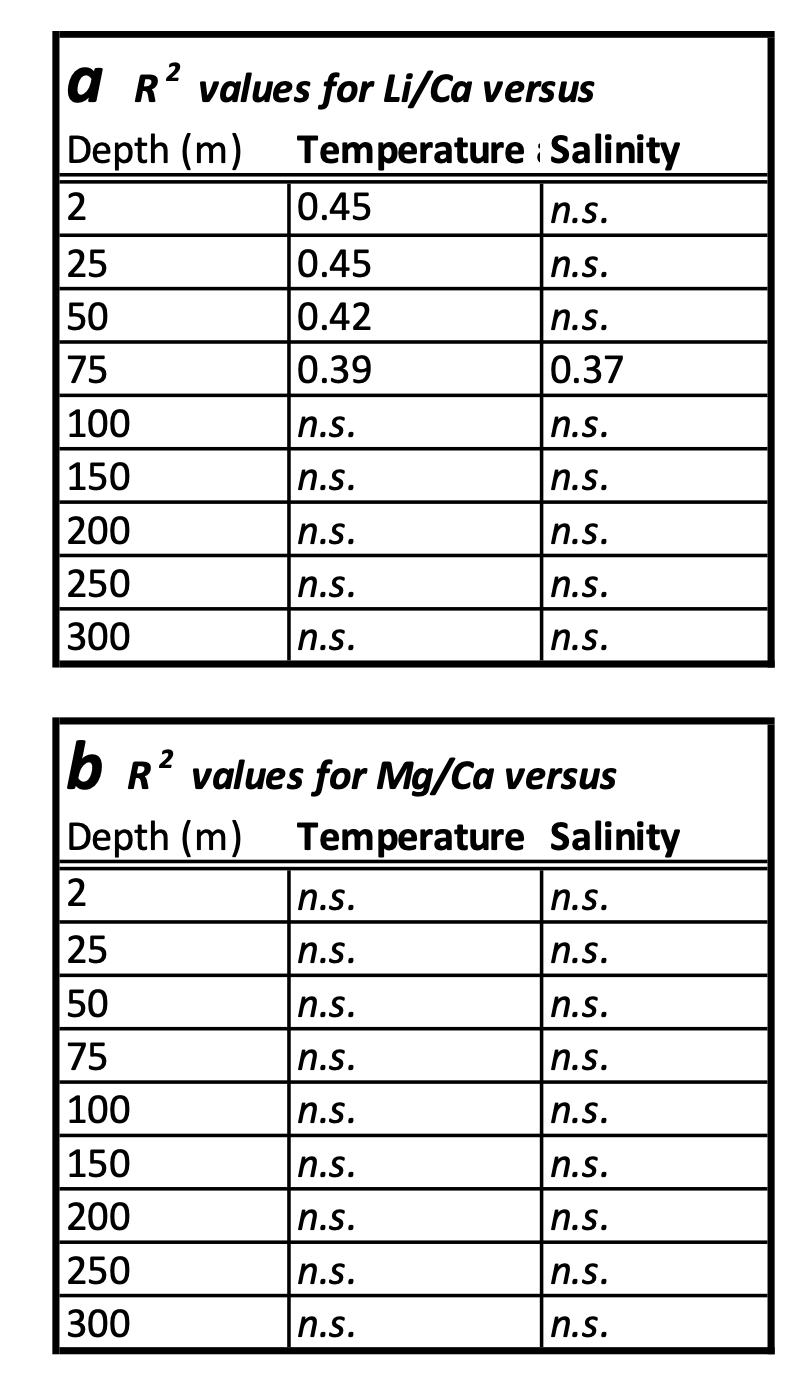


**Table S2.** **Statistical parameters for linear correlation** between (**a**) average pteropod shell Li/Ca (μmol/mol), (**b**) average pteropod shell Mg/Ca (mmol/moland selected water parameters: temperature (° Celsius) and salinity. Regressions were performed against parameters at specific depths; the R^2^ is reported when p < 0.05. *n*.*s*. indicates non-significant regressions (p > 0.05).

**
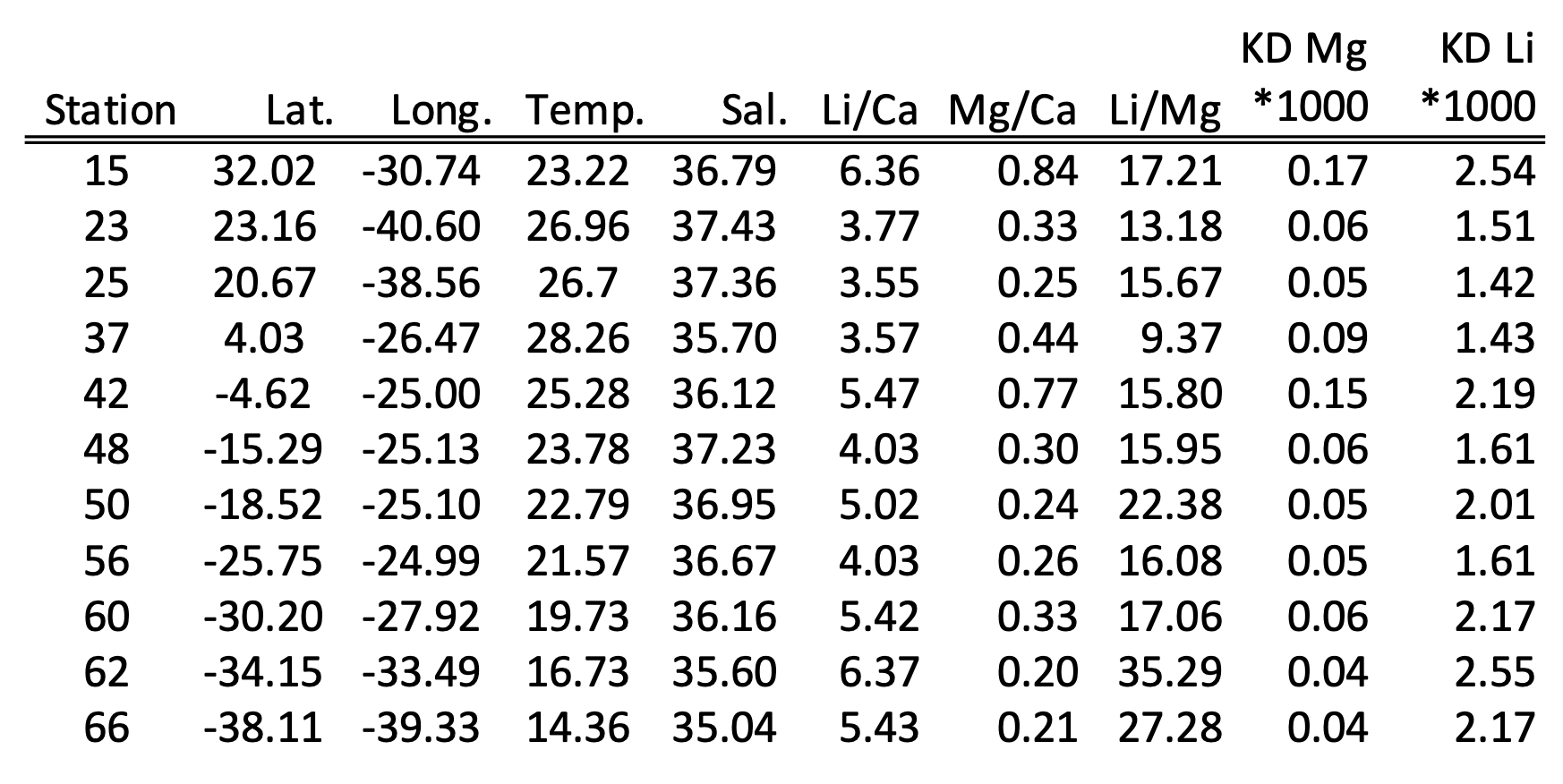
**

**Table S3: Partitioning coefficients for Li (K_DLi_) and Mg (K_DMg_).** Depicted are: station location information (Lat.= Latitude, Long= Longitude), Temperature at 50m depth (Temp., in °C), Salinity at 50m depth (Sal.) and average trace elemental composition (Li/Ca in μmol/m{Lebrato, 2020 #410@@author-year}ol, Mg/Ca in mmol/mol, Li/Mg in μmol/ mmol) with the partitioning coefficients for Li (K_DLi_) and Mg (K_DMg_). For the calculation of K_DLi_ and K_DMg_ seawater values of 5.1 mol/mol for Mg/Ca (Lebrato et al., 2020) and 0.0025 mol/mol for Li/Ca were used (Hathorne et al., 2013).


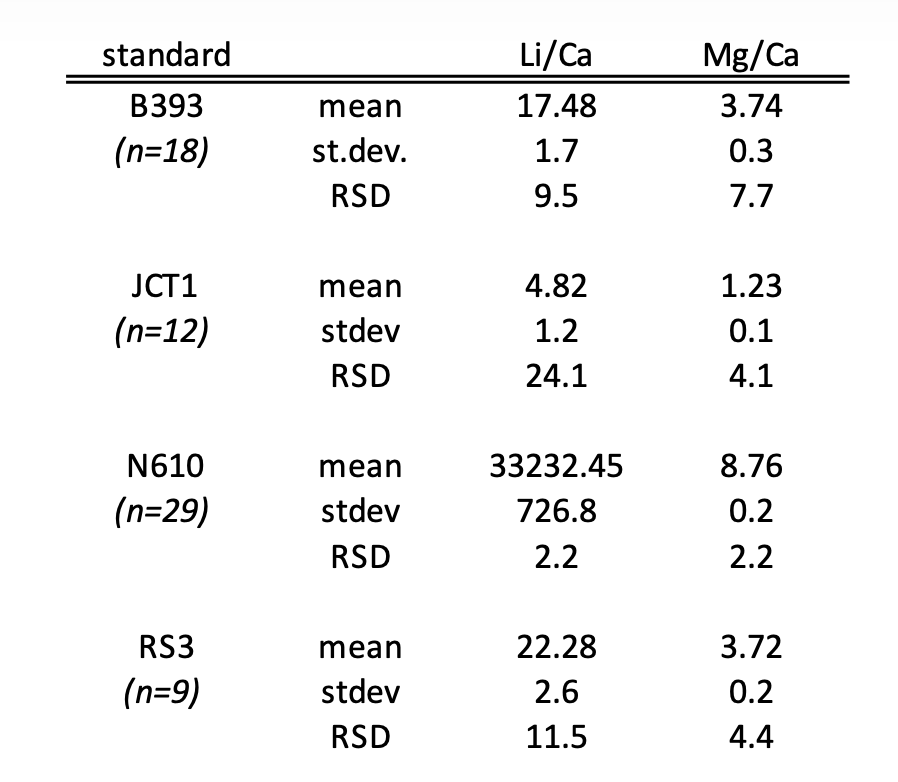


**Table S4: Trace elemental composition of standards.** Four standards (B393, JCT1m N610, RS3) were measured throughout the measurement period. Depicted are average trace elemental composition (Li/Ca in μmol/mol, Mg/Ca in mmol/mol) with their standard deviation (st.dev) and RSD (relative standard deviation, in %).

**References**

Hathorne, E. C., Felis, T., Suzuki, A., Kawahata, H. & Cabioch, G. Lithium in the aragonite skeletons of massive Porites corals: A new tool to reconstruct tropical sea surface temperatures. *Paleoceanography* 28, 143–152, doi:[10.1029/2012PA002311](https://doi.org/10.1029/2012PA002311) (2013).

Keul, N. *et al.* Pteropods are excellent recorders of surface temperature and carbonate ion concentration. *Scientific Reports* **7**, 12645, doi:10.1038/s41598-017-11708-w (2017).

Lebrato, M. *et al.* Global variability in seawater Mg:Ca and Sr:Ca ratios in the modern ocean. *Proceedings of the National Academy of Sciences* **117**, 22281–22292, doi:10.1073/pnas.1918943117 (2020)
